# A prospective clinical cohort-based study of the prevalence of OCD, obsessive compulsive and related disorders, and tics in families of patients with OCD

**DOI:** 10.1186/s12888-022-03807-4

**Published:** 2022-03-17

**Authors:** Lior Carmi, Vlasios Brakoulias, Oded Ben Arush, Hagit Cohen, Joseph Zohar

**Affiliations:** 1 Chaim Sheba Medical Center, Post Trauma Center, Ramat-Gan, Israel; 2 Israeli Center for the Treatment of Obsessive−Compulsive and Related Disorders, Modiin, Israel; 3 Western Sydney Obsessive−Compulsive and Related Disorders Service, Western Sydney Local Health District – Mental Health Services, North Parramatta, Australia; 4 School of Medicine and Translational Health Research Institute, Western Sydney University, Sydney, Australia; 5 Ministry of Health, Anxiety and Stress Research Unit, Faculty of Health Sciences, Beer-Sheva Mental Health Center, Ben-Gurion University of the Negev, Be’er Sheva, Israel

**Keywords:** OCRD, OCD, Prevalence, Family members

## Abstract

**Background:**

The lifetime prevalence of obsessive − compulsive disorder (OCD) is currently estimated at 2 − 3% and the prevalence in first-degree family members is estimated to range between 10 and 11%. Separating OCD from other anxiety disorders and including it into the new “obsessive − compulsive and related disorders” (OCRDs) category has had a dramatic impact on the diagnosis, while also contributing to the better understanding of the genetics of these disorders. Indeed, grouping OCD with body dysmorphic disorder (BDD), and body-focused repetitive behaviors such as trichotillomania (hair pulling), onychophagia (nail biting), and excoriation (skin picking) into the same diagnostic family has resulted in a much greater lifetime prevalence (> 9%). These diagnostic changes necessitate an updated epidemiological study, thus motivating this investigation.

**Methods:**

The study sample comprised of 457 patient’s cases from an Israeli and an Australian OCD center. Interviews were completed as a part of the intake or during treatment in each of the centers. Prevalence of OCD, OCRDs, tics, and other psychiatric comorbidities in first- and second-degree relatives was assessed by interviewing the OCD patients. Interviews were conducted by at least two researchers (LC, OBA, JZ) and only family information on which the interviewers have reached consensus was considered.

**Results:**

Initial analyses revealed an increase of OCD and OCRD prevalence in first- and second-degree family members as compared to the current literature due to reclassification of these disorders in DSM-5.

**Conclusion:**

The new category of OCRD has changed the landscape of epidemiological studies in OCD. Further and broader studies are needed in order to better understand the lifetime prevalence of OCRD in first- and second-degrees family member.

## Background

OCD is a global phenomenon, with a substantial similarity across cultures in symptom clusters, gender distribution, age of onset, and comorbidities [1]. Using the restricted DSM-5 definition (i.e., not including OCD-related disorders), the lifetime prevalence of OCD in the general population was calculated at 2–3% in several studies [2, 3]. Those percentages have been confirmed across cultures [4] and are supported the biological basis of OCD. Although the cultural, economic, and social factors play a role in the presentation of OCD (that is, the content of obsessions and the shape of the compulsions), its prevalence is equally distributed across the globe.

Although several approaches have been used to evaluate the role of heredity in OCD, twin and family studies remain the most common, as they allow comparisons between concordant monozygotic twins and discordant monozygotic twins with OCD, as well as familial aggregation. Based on their literature review, Van Grootheest and colleagues estimated that OCD is inherited in 27% to 47% [5] while childhood-onset OCD has an even greater genetic component (45–65%) [6–8]. The role of genetics in OCD has also been investigated by establishing OCD prevalence in family members of OCD patients. Numerous family studies have demonstrated the familial genetic contribution of OCD incidence. While the frequency of OCD and subclinical OCD differed within families across studies, the overall conclusion was that OCD and subclinical OCD are familial [9–14].

In accordance, relatives of OCD patients are twice as likely to develop OCD than those of healthy subjects, while rates in relatives of children and adolescents with OCD showed a tenfold increase relative to controls [15]. Furthermore, based on their review and meta-analysis of the genetic epidemiology of OCD, Hettema et al. reported an aggregate odds ratio of 4, supporting the familial aggregation of OCD [16]. In other studies, OCD prevalence among relatives of affected individuals was significantly higher than either the estimated population prevalence or rate among controls [17–20]. Significantly higher percentage of OCD cases was also reported by Grabe and colleagues in relatives of affected individuals based on both clinical and a general population study [21].

Tourette syndrome (TS) is a childhood-onset, neuropsychiatric disorder defined by multiple waxing and waning motor and phonic tics [14]. A related diagnosis, chronic tic disorder (CT), is characterized by persistent motor or phonic tics. Community surveys, conducted in various countries over the past twenty years provide estimates of prevalence for TS ranging from 0.5 and 38 cases per 1000 children [22]. Tourette syndrome/CT and OCD overlap in their phenomenological features, often cluster in families, and co-occur in affected individuals. About 30% of OCD patients have comorbid lifetime tic Disorders [23], and about 20% of TS/CT patients will suffer from OCD as well [24].

In DSM-5, OCD was separated from the anxiety disorders and was categorized within a new diagnostic category (denoted as ‘Obsessive–compulsive and related disorders’ or OCRDs), which includes, in addition to OCD, hoarding disorder, body dysmorphic disorder, and body-focused repetitive behaviors such as trichotillomania (hair pulling), onychophagia (nail biting), and excoriation (skin picking) [25].

Considering this spectrum-oriented view of OCRDs [26], its cumulative prevalence is estimated to be higher than that of OCD and can reach up to 9.5% [25]. The diversity of disorders subsumed under this new OCRDs category warrants an epidemiological study that takes these conceptual changes into account.

## Method

The study took place between September 2020 – February 2021. Cases of OCD patients from the Israeli Center for OCD and Nepean Hospital, Sydney, Australia were evaluated in this study. Interviews were completed as a part of the intake or during treatment in each of the centers. All patients met the criteria for OCD and scored > 15 on the Yale-Brown Obsessive Compulsive Scale (Y-BOCS) [27].

### Diagnostic Procedure

The study was approved by both IRBs; The research Ethics Committee at Chaim Sheba Medical Center (‘International Review Board for human and animal trials’- No. 7204–20-SMC) and the Western Sydney Local Health District Human Research Ethics Committee at Nepean Hospital (‘Human Research Ethics Committee’ No. 2019/ETH01314). All subjects were interviewed by clinicians (psychiatrists or doctorate-level clinical psychologists) who had undergone extensive training in the diagnostic procedure.

In the Israeli cohort, demographic data, OCD domains, presence of other OCRDs or other comorbidities, as well as information on current and past tics was gathered during the interview, which also probed into the presence of OCD or OCRDs, other comorbidities, and current and past tics among family members and relatives. For the purpose of analyses, parents, children, and siblings were considered family members, whereas grandparents, uncles, aunts, cousins, etc. were considered second-degree relatives. In the Australian center, a structured clinical assessment was conducted using Mini Neuropsychiatric Diagnostic Interview, Y-BOCS, Shapiro tic severity scale, and Family history screen. However, patient’s OCRDs comorbidities were not evaluated.

#### Statistical analysis

Data analysis was performed using the IBM SPSS 25 software. Differences between the OCD sub-groups (i.e., Israeli vs. Australian patients; patients with vs. those without tics; individuals with vs. those without OCRD comorbidity) and the prevalence of OCRDs in the family were evaluated using a chi-squared test for independence (Mantel–Haenszel chi square), with Group (OCD patients with and without tics or OCRDs) as the independent variable and OCRD incidence in the family as the dependent variable. Mean differences in age were analyzed using t-test. The required *p*-value for significance was corrected (Pc) for the relevant number of comparisons.

## Results

The study sample comprised of 457 cases (222 recruited from the Israeli Center for OCD and 235 from Nepean Hospital in Australia). 103 patients were diagnosed with comorbidity, the main comorbidities were depression (*n* = 23), Social anxiety (*n* = 14), Addiction (*n* = 15). No statistically significant differences were found in sex (f/m) distribution (Israeli cohort: 108/114; Australian cohort: 124/111), severity score as measured by Yale-Brown Obsessive Compulsive scale at baseline (YBOCS, Israeli cohort: Mean: 28, SD: 3.3; Australian cohort: Mean: 26, SD: 2.9), or number of patients. The patients in the Israeli cohort were found to be younger (Mean: 25.3 years; SD: 11.6 years) than those in the Australian cohort (Mean: 43.1; SD: 15.8), t (455) = -13.6, *p* < 0.001.

### OCD and OCRD prevalence in family members

**Fig. 1:**
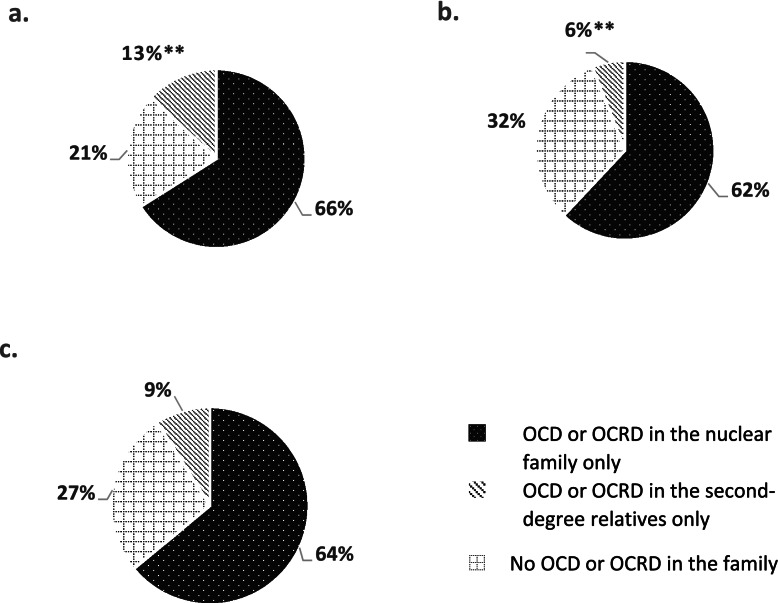
Percentages of OCD or OCRDs in family members. Percentages of OCD or OCRDs in the family of the Israeli (**A**) and Australian (**B**) patients, and general (**C**) cohorts. ** *p* = 0.003

Seventy three percent of the entire OCD cohort (335/457) had first- and second-degree family members affected by OCRDs or OCD. The most common disorders were: OCD (*n* = 197, 43%), Hoarding (*n* = 98, 21%), BDD (*n* = 47, 10%). No statistically significant difference was found in the OCRD prevalence in the nuclear families of Israeli and Australian cohorts (147/222 or 66% vs. 145/235 or 62%, *p* > 0.05). However, higher prevalence of both OCD and OCRDs in the second-degree relatives was noted in the Israeli cohort (30/222, 13%) relative to the Australian cohort (13/235, 6%, χ^2^ = 8.5, *p* = 0.003), as shown in Fig. 1. (Please see Table 1 for demographical characteristics).

**Table 1:** Demographic and clinical characteristics of the OCD patients sub-groups

	Patients with no OCD in the family (*n* = 122)	Patients with OCRCD\OCD First degree family member (*n* = 292)	Patients with OCRD\OCD second degree family member (43)	Significance
Age (Mean, SD)	26, ± 2.9	29, ± 3.2	36, ± 4.4	n.s
Sex (f/m)	66/56	157/ 135	25/18	n.s
YBOCS at baseline (Mean, SD)	29, ± 2.1	26, ± 3.1	28, ± 2.9	n.s

**Fig. 2:**
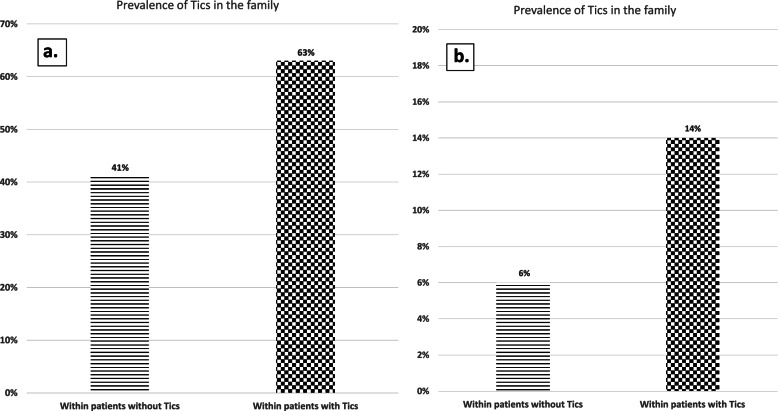
Family’s prevalence of tics of patients with and without tics. Prevalence of tics in family members (first- and second-degree) of patients with and without tics: (**A**) Israeli cohort, (**B**) Australian center. **A **Overall risk – 51%, risk difference – 22%, risk ratio – 1.5. **B** Overall risk – 7%, risk difference 8%, risk ratio – 2.4

Tic syndrome was identified in 133 (29%) patients, with significantly higher percentage within the Israeli cohort (105, 47%) relative to the Australian cohort (28, 12%; χ^2^ = 69.1, *p* < 0.0001). In the entire cohort, tic syndrome was identified in 130 (28%) family members (first- and second-degree). However, the prevalence of tics within the family (first- and second-degree) of patients with tics was significantly higher (50%) compared to patients without tics (19%, χ^2^ = 44.2, *p* < 0.001). This pattern was observed when the Israeli (63% vs. 41%, χ^2^ = 10.5, *p* < 0.001) and the Australian (14% vs 6%, χ^2^ = 2.7, *p* < 0.05) cohorts were analyzed separately, as shown in Fig. 2.

**Fig. 3:**
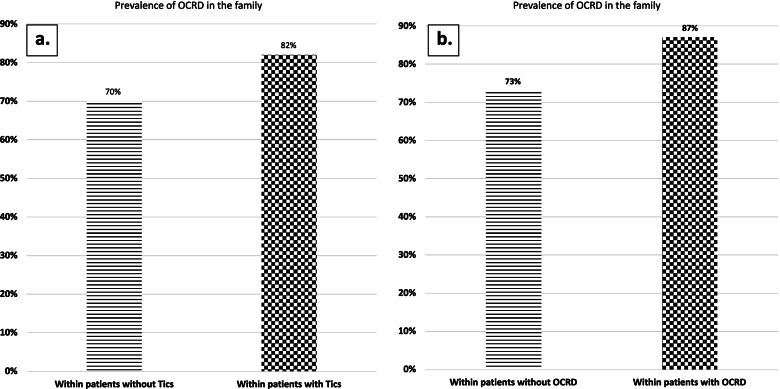
Family’s prevalence of OCRD of patients with or without tics. Prevalence of OCRD in the family of (**A**) patients with (*n* = 133) or without tics (*n* = 324) and (**B**)*Only within the Israeli cohort, segregated into patients with (*n* = 93) and without OCRD comorbidities (*n* = 129). **A** Overall risk – 73% (CI: 69 − 77.1%), risk difference – 12.2% (CI: 4 − 20%), risk ratio – 1.17 (CI: 1 − 1.3). **B** Overall risk – 79.2% (CI: 73.4 − 84.1%), risk difference – 13.4% (CI: 3.2 − 23.6%), risk ratio – 1.2 (CI: 1 − 1.3)

In addition, OCRD prevalence in the family (first- and second-degree) of patients with tics (χ^2^ = 7.1, *p* = 0.003) and other OCRD comorbidities (χ^2^ = 5.9, *p* = 0.007) was higher compared to patients without tics or OCRD as shown in Fig. 3.

## Discussion

In accordance with the inclusion of OCD as a part of the OCRD chapter in DSM-5, we evaluated the OCRD prevalence in family members of OCD patients in Israel and Australia. Our analyses revealed that 73% of the cohort had a first- or second-degree family member with an OCRD. Although higher than previous reported prevalence, former studies (focused only on OCD and Tic disorder) have also found a significant genetic contribution both in Tic and Obsessive- compulsive disorders and a shared genetic architecture [14, 28].

While the OCRD prevalence within the nuclear family was similar between the sites, the prevalence within second-degree relatives was higher in the Israeli cohort relative to the Australian sample, which can be attributed to the difference in the number of family members in these countries (according to https://data.oecd.org/pop/fertility-rates.htm, fertility in Israel is 3.1 vs 1.7 in Australia). It is also likely due to the younger Israeli cohort, and thus perhaps a greater number of second-degree relatives who are still alive. The discrepancy in findings could also be explained by the differences in the data collection process, as structured interviews were conducted at the Australian site, whereas Israeli patients were subjected to a detailed interview. It should also be noted that the Australian site was located in an area of Sydney where many migrants live and work, to the extent that approximately 50% of the population was born overseas. As a result, patients that were recruited for the study may not have seen their relatives for many years and may not be aware of OCD symptoms or tics in their relatives.

Increased prevalence of tics was also noted in the Israeli cohort relative to the Australian subjects, which could again be ascribed to younger age and differences in data processing methods but also to the higher family aggregation and endogamy [29]. Specifically, tics tend to affect younger individuals more than older patients [30], and would thus be more prevalent in the Israeli cohort. It is also worth noting that, in the Australian center, data regarding tics was obtained via structured interviews and self-report questionnaires, while this information was gathered during a clinical interview at the Israeli center. Active assessment and observation for tics in the Israeli cohort may have led to an increased potential for detecting tics, resulting in more accurate prevalence. Indeed, the prevalence of tics within the Israeli cohort (47%) is in line with the results yielded by extant studies (e.g., [23]).

Nearly 30% of the cohort had a family member that suffered from tic disorder. This is in line with former studies that have shown high prevalence of comorbidity and with comorbidity likeliest to occur in their childhood-onset forms [11, 31]. More recent study has shown common neurological basis of both disorders [32]. Specifically, Converging evidence from animal studies and neuroimaging studies suggests that dysfunction in cortical basal ganglia circuitry mediates tics and compulsive behaviors, supporting a role for shared biological vulnerability [33, 34]. Accordingly, the prevalence of tics or OCRD in the family was significantly associated with the patient’s condition and was significantly higher in families of patients that also suffered from tics or OCRD. As this pattern was observed in both Israeli and Australian sample, it may imply a meaningful contribution of genetic loading in OCRD and tic disorder. These results are congruent with the findings yielded by prior family studies in which OCD symptoms were more frequent among family members of patients diagnosed with Tourette’s syndrome (TS) [35]. Leonard et al. also noted higher TS and tic prevalence in first-degree relatives of children with OCD [11]. This is also in line with previous studies, that although focused on OCD per se (and not OCRD), have found that genetic factors play an important role in its etiology, particularly in patients with comorbid tic disorder [36, 37].

When interpreting these findings, several study limitations should be noted. First, the data collection process was not harmonized across the two centers. Specifically, while the data from the Australian center was collected through validated structured assessments and questionnaires, in the Israeli center, a clinical interview was conducted with the patients. Second, as none of the family members were interviewed, the prevalence reported in this manuscript is based on patient accounts and may relate to OCD symptoms rather than a formal diagnosis. Finally, the time of onset of the disorder (and specifically early onset) is found to be related to Tics comorbidity. However, this data was not assessed in the entire cohort and therefore was not analyzed.

## Conclusion

In conclusion, there appear to be high rates of familial prevalence in OCD that are further increased by the presence of tics and OCRD. Further studies are thus required to assess the genetic relationship more accurately between OCD and tic disorder.

## Data Availability

All data is available upon request.

## Abbreviations

OCD: Obsessive compulsive Disorder
OCRD: Obsessive compulsive and related disorder.
TS: Tourette syndrome
CT: Chronic Tics
